# Synthesis and Validation of a Weatherproof Nursery Design That Eliminates Tropical Evening-Fever Syndrome in Neonates

**DOI:** 10.1155/2014/986760

**Published:** 2014-02-18

**Authors:** Hippolite O. Amadi, Lawal I. Mohammed, Mohammed B. Kawuwa, Abdulquddus Oyedokun, Hajjah Mohammed

**Affiliations:** ^1^Department of Bioengineering, Imperial College London, SW7 2AZ, UK; ^2^Neonatal Unit, Department of Paediatrics, Federal Medical Centre Nguru, Nigeria; ^3^Department of Obstetrics and Gynaecology, Federal Medical Centre Nguru, Nigeria

## Abstract

Neonatal thermal stabilisation can become challenging when uncontrollable factors result in excessive body temperature. Hyperthermia can rapidly slow down baby's progress and response to treatment. High sunlight intensity in tropical countries such as Nigeria manifests in incessant high neonatal temperatures towards early evenings. The ugly consequences of this neonatal evening-fever syndrome (EFS) can only be eradicated by the development of a controlled weatherproof nursery environment. Two laboratories and a ‘control ward' were applied. Lab-2 was a renovation of an existing room in a manner that could correct an existing nursery. Lab-1 was an entirely new building idea. The laboratories were assessed based on comparative ability to maintain environmental coolness and neonatal thermal stability during hot days. Data collection continued for 12 full calendar months. On average, at evaluated out-wind peak temperature of 43°C (range: 41°C–46°C), the control-ward peak was at 39°C, Lab-2 peak at 36°C, and Lab-1 peak at 33°C. All incubators in the control overheated during the hot periods but there was no overheating in Lab-1. Forty-four (86%) of sampled babies were fever-quenched by water sponging 131 times in the control whilst only one baby received same treatment in Lab-1. Nursery designs patterned after Lab-1 can significantly reduce EFS-induced neonatal morbidity.

## 1. Introduction

Literature has identified and defined evening-fever syndrome (EFS) as the thermal capacitance effect of overheated buildings that manifests in neonatal hyperthermia usually during the mid afternoon and early evening periods of sunny days [1]. EFS affects all newborns, especially the premature escalating morbidity, prolonging hospitalisation period and impoverishing overall outcome. The effect of EFS has been reported from many parts of Nigeria with extreme cases occurring within the Savannah regions of the middle-belt and the desert northern regions of the country [2]. At the Federal Medical Centre Nguru (FMC-Nguru) located within this zone, EFS is a significant contributor to neonatal morbidity resulting from soaring local ambient temperature that goes as high as 47°C at times. In a recent publication Amadi et al. (2013) carried out a study to characterise EFS [3]. This implicated the climate, environment, and civil structures among other reasons for excessive room warming that instigates EFS. This suggested that EFS is a condition of time-dependent neonatal hyperthermia that can be clinically diagnosed, typically being associated with the following constituents:

- it notably occurs on high ambient days, often in excess of 37°C
- it readily affects neonates within room/nursery building of which walls, windows, and/or ceiling are directly exposed to high intensity of rays of sunlight
- neonates acquire high body temperature during early postmeridiem (p.m.) perhaps due to heat transfer from the energised surroundings but regain thermal stability much later in the evening when the surroundings have cooled down.

Apart from occasional planting of trees within residential compounds in Nguru town and the raising of concrete flooring by up to 1 ft above ground level as seen from the structures at FMC-Nguru, there are no other building standards that technically suggest adaptation to this extreme high ambient temperature. As a result the interiors of buildings including the Special Care Baby Unit (SCBU) of this hospital often get higher than 40°C of room temperature. This is significantly high enough to trigger hyperthermal crisis for the immature neonate even for a short period of exposure [4, 5]. Neonatal thermal stability, defined as the condition of maintaining the body temperature of a given neonate within the physiologically allowable range of 36.5°C to 37.4°C, is a crucial factor for the survivability of any newborn.

The SCBUs of Nigerian hospitals often respond to this extreme condition of environmental high temperature with all kinds of desperate clinically inefficient measures. Some clinicians often suspect a disease process responding with doses of unnecessary antibiotics and complicating situations and from which many neonates might have lost their lives. In addition, nurses resort to the desperate practice of opening incubator access windows/doors, turning-on fans, turning-on air conditioners or entirely switching-off the incubator. At the FMC-Nguru babies were sponged down in flowing water when body temperatures reached 37.9°C. There are down-sides to these desperate measures: opening port-holes compromises the microenvironment as a mini-isolation unit for the delicate premature baby [6, 7]; fans and air conditioners are subject to adequacy of power supply, frequent breakdown, and threat of hypothermia for cot babies [8]; incubator switch-off often results in sudden hypothermia during environmental thermal recovery at sunset; baby bathing has momentary effect only and often demands unaffordable high volume of nursing time as this has to be repeated several times during the day. This hence results in frustration for the few nurses that would attempt to thermally stabilise any neonate.

We hypothesise that any measures that can achieve naturally cooled nursery environment would eliminate the uncontrollable excessive warming of neonates due to EFS. The relatively poor setting of tropical Africa would constitute complications due to factors that are naturally difficult to change or be sustainably managed. These involve the sun and sunlight intensity as the source of heat generation, the relocation of the hospital complex to a more appropriate site, provision of uninterruptable air conditioning units, or supply of specially designed adaptable incubators to replace a host of the climate-stricken standard models. In the face of these seeming difficulties it became imperative to seek to investigate how design or building structural factors could be optimized for a probable permanent solution [9, 10]. Therefore, the aims and objectives of this study were (1) to develop miniaturised laboratories with distinct unconventional building factors that are capable of ameliorating the warming effect of the sun in a nursery and (2) to validate these by carrying out a comparative analysis of room warming and neonatal thermal stability of babies being nursed in the laboratories.

## 2. Materials and Methods

The project was divided into two segments involving (1) design and construction of two different nursery laboratories and (2) validation of the new constructs through “controlled” comparative analyses of outcomes. Ethical approval for the execution of the project was obtained from the Medical Ethics Committee of the Federal Medical Centre Nguru, Nigeria. Patient-informed consents for the validation aspect of the research were also obtained from carers, in most cases the mothers, after carefully explaining the project and how their babies might be minimally involved in the use of data collected during normal nursing/clinical procedures whilst treating them. It was more difficult recruiting from illiterate carers who were more in number than the educated ones. A total of 97 neonates were recruited, 33 for the wet season months (September-October 2012) analyses and 64 for the dry season months (March-April 2013) analyses. Average admission-weight was 1966 g (range: 1050 g–2500 g). Average gestation age of the sample population could not be accurately quantified as most cases lacked antenatal information.

### 2.1. Laboratory Designs and Construction

Literature reveals that nursery warming due to extreme sunlight intensity comes through “capacitance” effect of nursery walls that are directly heated by the sun amongst other mediums [3]. This was enlisted as the major design constraint for the new application. The laboratory design therefore incorporated a double-wall with in-between air space for all sides that were exposed to direct rays of sunlight. Other probable mediums of sun-heat transmission were individually assessed. Parameters that could possibly ameliorate excessive heat transfer were carefully integrated into the design. These were flooring, roof/ceiling height and materials, window size and positioning, window blind materials, and room lighting. Two different laboratories were designed and constructed. The first (Lab-1) represented the construction of a “purpose” built nursery structure that targeted the total elimination of EFS. This integrated all possible constraints that were capable of minimising the transmission and concentration of sun-heat within the interior of the structure. The second laboratory (Lab-2) represented a possible renovation that could be done to an existing nursery structure so that the warming of the interior by the sun would be considerably reduced.

Lab-2 was designed to avoid expensive or extensive constructions that could be either unaffordable or lengthily disruptive to the activities of an existing nursery. This was implemented by the renovation of an existing office space at the Special Care Baby Unit (SCBU) complex of FMC-Nguru. The double-wall for the side exposed to sunlight was provided by adding an inner wall on that side. An arbitrary air space of 6 cm was provided in-between the walls and vented into the roof space through the ceiling. This created escape route for heated air within the space and was expected to enhance the reduction of heat transfer to the inner wall. Lab-2 was finished to a rectangular floor area of 5.9 m^2^, the original ceiling height of 3 m, and one door and one window.

The design of Lab-1 extensively integrated other measures that could contribute to further heat reduction on the inner wall and the interior of the structure as explained below.

**Figure 1:**
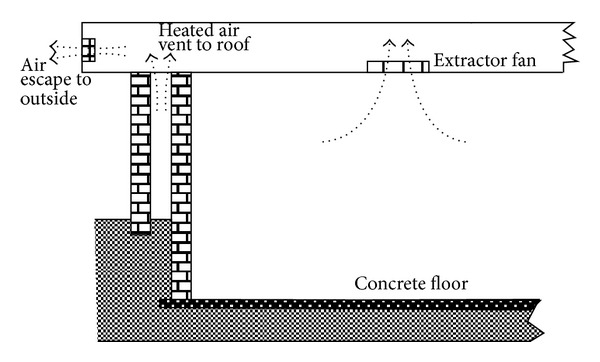
Cross-section through Lab-1.

**Figure 2:**
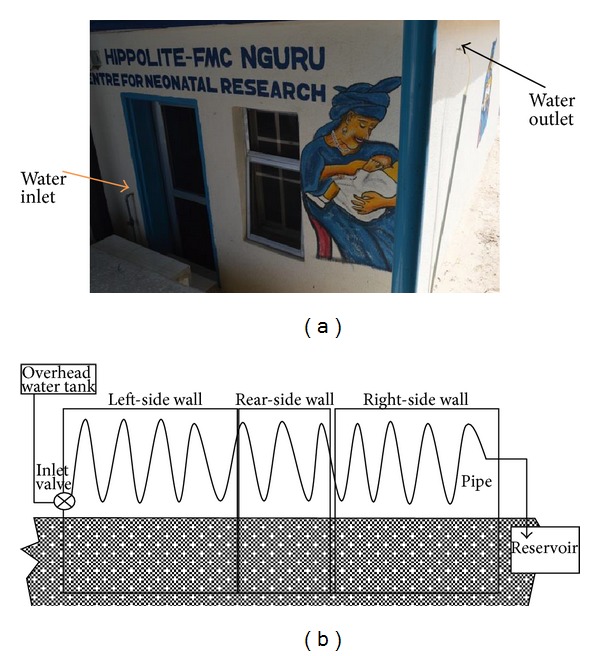
(a) Lab-1 showing heat exchanger water inlet and outlet. (b) Heat exchanger copper piping through the walls of Lab-1. Overhead tank delivered water through an inlet valve. Circulation was ensured automatically through the positive pressure head created by the relative height of the overhead tank. Cool water in the under-surface reservoir was pumped back to overhead tank when required.

1. Prior to design, a simple thermometric investigation revealed that a significant temperature difference existed between the sandy ground level surface of the site and the bottom of an excavation 90 cm below the surface. It was therefore considered a good idea to take advantage of this and tap the coldness for the room. The Floor was therefore designed as a 5 cm thick concrete mixture that was set at 120 cm below ground level. The surrounding single wall continued until ground level where this transformed externally into the double wall as described for Lab-2. Inner walls and floor were finished in smooth ceramic tiles.
2. The floor-to-ceiling height was 380 cm, that is, 80 cm more than the standard height of rooms in the hospital complex. This was to achieve a good height for warmer air that tends to raise the room temperature at the height of the cots and incubators. A roof extractor fan that automatically covered its outlet whilst switched-off was provided to maintain the escape of heated air from the room and vented air within the roof space as shown in Figure 1.
3. A controlled valve-operated heat exchanger was integrated in the design. This was a system of flowing water made to traverse the interior surface of the inner wall, behind the tiling. The copper-piped water entered building from the left end via a manual valve, crisscrossed the entire upper half of the inner wall above ground level, and exited through the right end (Figure 2(a)). Water was supplied from an insulated overhead tank to ensure adequate pressure head and emptied into the same underground tank from which the overhead tank received supply (Figure 2(b)). The underground water storage served a purpose of recooling the water before recycling to the overhead tank. Heat exchanger was operated during data collection on few prescribed days only for the investigation of any possible contribution such application might offer towards the cooling of Lab-1. The coolness pattern of Lab-1 relative to heat exchanger days was evaluated by the quantification of average temperature rise of the water from the system outlet as a measure of heat extraction from the Lab interior.
4. Two opposite wall windows were integrated such that these were indirectly facing each other to ensure better air circulation. Cotton material window blinds were affixed and operated when sun-heat intensity through the windows became very intense. Electric lighting of the interior and medical examination lamps were provided by low wattage LED system installations to minimise heat generation by these applications.

Lab-1 was finished to a rectangular floor area of 8.3 m^2^, roof height of 3.8 m, and one door and two windows.

### 2.2. Experimental Design and Data Collection

The existing nursery section for babies born within the FMC-Nguru facility (In-born Unit) was applied in the study as the “control ward” while Lab-1 and Lab-2 were the test-wards. The structural architecture of the control ward remained unaltered. This has a rectangular floor area of 70.5 m^2^, room height of 3 m, and two opposite French doors and six windows arranged for effective crossventilation with window-blinding shutters.

Electric power supply to all three wards was provided via two assembles of 3.5 KVA inverter “power-banks” (Fusion Series, Su-Kam industries, India). The power-banks ensured uninterrupted power supply to the experimental wards all through the periods of data collection. These acted as ready backups during unexpected power failures as normally witnessed in Nigerian cities. Environmental temperature, humidity, and other meteorological data within all three wards and the outside of the complex were automatically collected and stored every 30 minutes. This was achieved by the application of three assembles of Watson's W-8681 Weather Station. Collected data were regularly downloaded to a standby computer for postprocessing.

Babies from consenting carers/parents were randomly assigned to available cots and incubators in all three wards. Experimental setup ensured a minimum of 1.5 sq. meter area per incubator and 1 sq. meter area for a cot in all sections. Neonates were nursed and vital signs data were collected by nurses at the usual practice times of the day. Nurses were posted to the wards on rota basis and the FMC-Nguru standard neonatal nursing techniques and procedures particularly remained unaltered in the three wards. This also involved the practice that required any hyperthermic baby to be water-bathed (sponged) if body temperature climbed up to 37.9°C. Exclusion/inclusion criteria were carried out as follows:

- It included all preterm neonates during special care nursing.
- It included all full-term neonates below 2500 g birth weight but was limited to half of the neonatal period (first 2 weeks of life) as healthy older neonates might be capable of significant auto thermal regulation [11].
- It excluded any neonates undergoing treatment for a well-established disease or disease process.

Experimental setup and data collection continued for over 24 months, covering every month of the year for up to two times. Data for the latest 12 months were applied for the comparative analyses of outcomes from the three wards in relation to the impacting environmental conditions of the outside surrounding.

### 2.3. Data Analysis

The collected data was applied to study impact of extreme hot days in the laboratories and the control ward. The hotness of a day is defined by its maximum outdoor wind temperature. This was treated as an extreme hot day if the day's peak temperature exceeded 36°C. The entire data covering the 12 months of the year was carefully examined, extreme hot days were identified, and their data were extracted into a file and were applied for further processing as described below.

- Plots of temperature against time-of-the-day were produced in superimposition for all three wards and outdoor temperature. These were produced for each extreme hot day to study the daily pattern of heat gain or loss in each ward in relation to changes in out-wind temperature.
- Records of relative-humidity (RH) against time-of-the-day were studied to assess the suitability of the wards for effective neonatal nursing without extra room humidification. This was also used to determine how the wards responded to changes in outdoor RH.
- The daily peak temperatures for each of the four environments (i.e., the 2 labs, the control ward and outdoor environments) for all extreme hot days were identified and their averages for each environment quantified.
- The averages of the daily relative humidity were quantified for each of the four environments.
- The behaviour of incubators in all three wards on the extreme hot days under assessment was evaluated from an incubator data collection chart to determine how environmental overheat affected them.
- All the patients' temperature chart folders for the period of study were recalled. Patients admitted during the two hottest months of the dry season (March and April) and the two relatively hot months of the wet season (September and October) were extracted and applied to obtain the total number of baby water-sponging events for each of the three wards being studied.
- The effect of the heat exchanger was evaluated by activating this on other specific days primarily to test the exchanger only. Temperature of water at laboratory inlet and outlet were measured and noted at 12 noon, 3 pm, and 6 pm on each day. Data was applied to quantify the averages of inlet and outlet water temperatures during the specified times of the day. This would enable the evaluation of the most cost-effective time of operating such a device to improve heat extraction from the ward.

### 2.4. Costing

The working floor areas of the control ward and labs were not equal in dimension to enable a direct building cost comparison. However, engineers were commissioned to design and produce bill-of-quantities for 2 equal size structures of floor area of 16.6 m^2^, each based on the construction details of the control ward and Lab-1, respectively. They also separately quantified the cost of adding double-wall to the two sides of the control ward patterned design. This served for the cost implication of renovation as in Lab-2. All quantifications were based on the prevailing costs of building materials and labour within and around the town of Nguru Nigeria. This revealed that the sum of 4.69 million Naira (about US$29,000) was required to complete the project based on the “control”, including all fittings and air conditioning. Lab-1 patterned counterpart was estimated to cost 4.93 million Naira (about US$31,000) including entire heat exchanger assembly. Extra double wall on two sides for Lab-2 pattern would require NGN317,000 (about US$2,000).

## 3. Results

**Figure 3:**
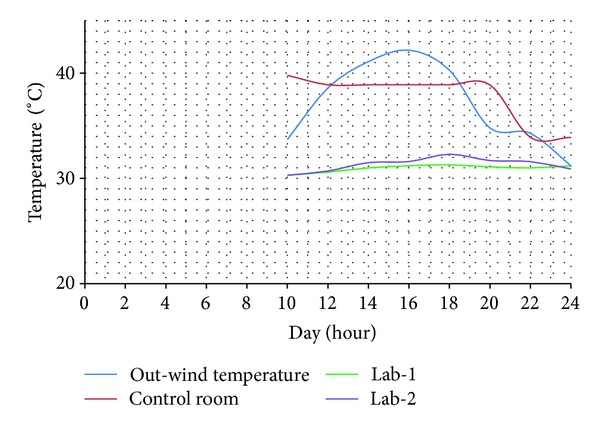
A typical day temperature chart during the wet season months of September and October. “Out-wind temperature” is the temperature of outside environment; “control room” is the ambient temperature of the control ward; “Lab-1” is the ambient temperature of Lab-1; “Lab-2” is the ambient temperature of Lab-2.

**Figure 4:**
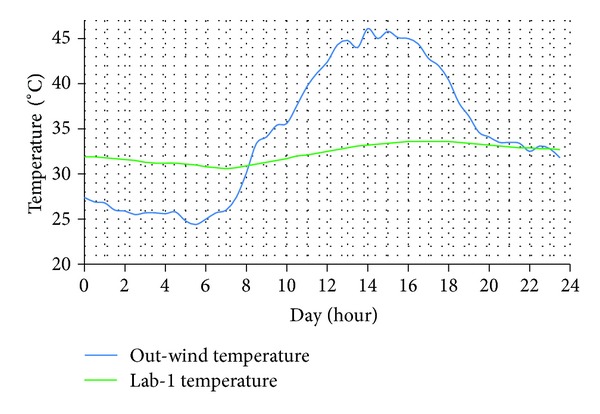
Overall hottest day. This was 7th April 2013 with a high temperature of 46.1°C. “Out-wind temperature” is the temperature of outside environment; “Lab-1” is the ambient temperature of Lab-1.

Superimposed daily plots of environmental warming for 24 hours of the day typically showed that ward peak temperatures were always lower than the out-wind peak temperature during the day times. These also revealed that Lab-1 peak temperatures were always the lowest and rarely exceeded 33.5°C even when out-wind peak soars up to 46°C (Figures 3 and 4).

On the contrary, the control ward always registered the hottest peak temperatures of the day amongst the three tested wards. This was typically lower than the out-wind peak temperatures, often within 3°C. On the average, Lab-1 was constantly much cooler than out-wind peak-temperature often with margins of up to 11°C (Figure 4).

**Figure 5:**
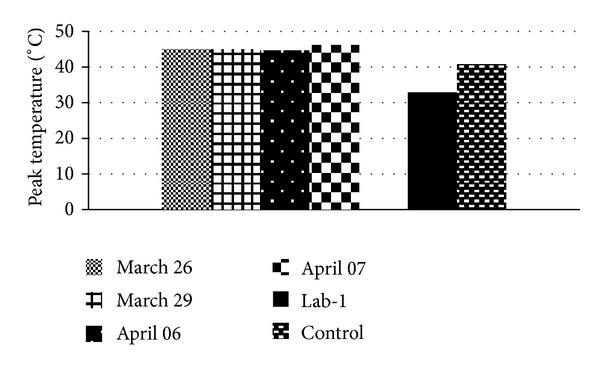
Out-wind peak temperatures for the four hottest days of the study period. “Lab-1” and “control” are the averages of the peak temperatures recorded in Lab-1 and control ward, respectively, during these 4 days.

When the four overall hottest days of the study period (each registering over 44°C peak temperature) were separately examined as shown in Figure 5, Lab-1 remained stably cool at 32.9°  ±  0.5°C; range: 32.1°C to 33.2°C across the four days.

**Figure 6:**
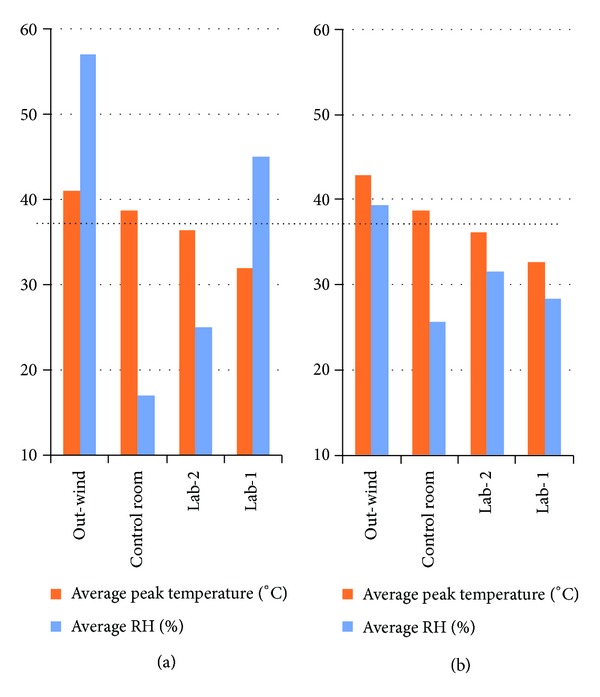
Peak temperatures and RH of respective environments, (a) wet season and (b) dry season. Broken line shows the critical threshold of 37.4°C above which neonatal hyperthermia could set in.

Figure 6 shows a chart of the overall average peak temperatures of the entire extreme hot days for the four environments during the extreme hot days of dry season months of March/April and wet season months of September/October with their average relative-humidity (RH).

**Figure 7:**
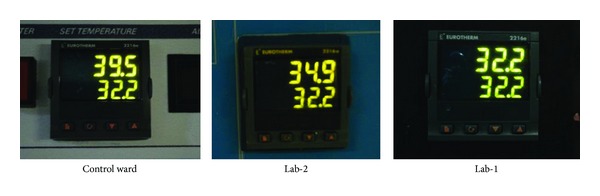
Incubator performance on typical extreme hot day of October 17, 2012. Readout top (actual chamber temperature) and bottom (set-point).

Typically, all the incubators in Lab-1 maintained set-point values at all times without overheating during the extreme hot days. The incubators of Lab-2 overshot set-point on few occasions while those of the control ward were constantly overheated beyond set-point values.  Figure 7 shows the typical performance of three incubators set at the same operational set-point value of 32.2°C in the three respective wards as captured on October 17, 2012.

**Figure 8:**
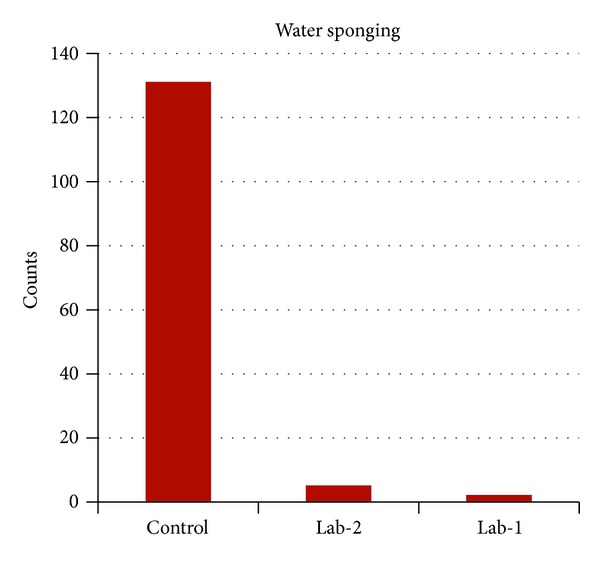
Total count of baby water-sponging events during the months of September, October, March, and April. There was only one sponging event in Lab-2 and none in Lab-1 during the September-October seasonal segment. Baby sponging occurred regularly in the control during both seasons.

Figure 8 shows the outcome of baby water-sponging events as a result of EFS-induced hyperthermia in the 3 wards for the months of March, April, September, and October. On the overall impact on the wellness of neonates, 70% (representing 28) of babies in the control ward were water-sponged 63 times during the wet season months of September and October. During the dry season months of March and April, 100% (representing all 16), participating babies in the control ward were sponged 68 times. Lab-1 folders revealed only 2 water-sponging events on 1 of 13 participating babies during the dry season months of March and April. There was no reported case of water sponging in Lab-1 all through the wet season study period. We observed that preterm and low-birth-weight (LBW) babies were quicker to succumb to EFS attack as compared to full term and bigger neonates. Full term babies were more susceptible within the first 7 days of life. Preterm and LBW babies were always under threat for most of the admission period. In addition to excessive high body temperatures, attack was found to be associated with relative increase in heart-rate (HR) most of the time. Respiration-rate (RR) was also affected as this was faster than normal in some patient but slower than normal in other patients during EFS attack. Water sponging would bring about sudden quench of fever for a short period of time. This improved RR in some patients for the short time but this did not necessarily slow down HR during this period.

**Figure 9:**
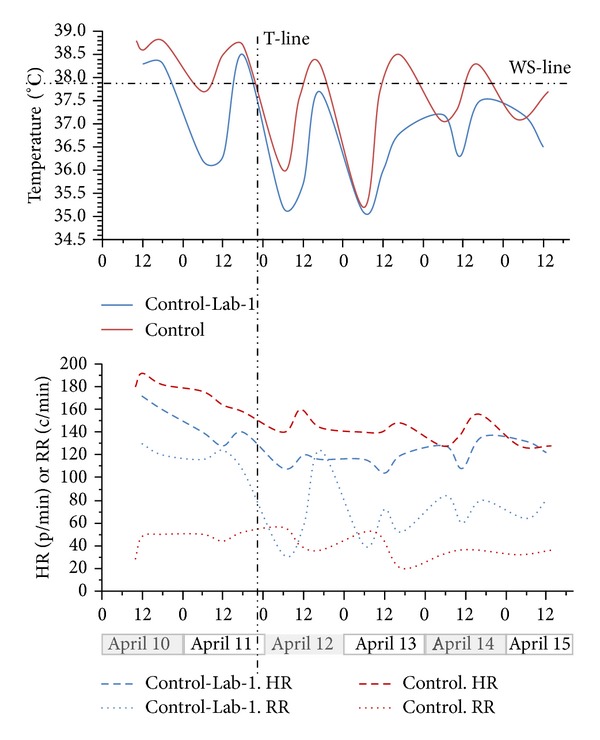
Comparative relationship between vital signs of two neonates nursed in the control ward and Lab-1. Baby “control” weighed 2250 g and spent all the indicated 6 days in the control ward. Baby “control-Lab-1” weighed 2450 g, admitted in the control ward but transferred to Lab-1 just before midnight on 11 April. HR is heart rate (pulse/minute). RR is respiration rate (cycle/minute). T-Line is the time at which “baby control-Lab-1” was transferred. WS-Line is the critical temperature above which baby must be water-sponged as practiced at FMC-Nguru.

Figure 9 is a graphical display of the signs of skin-temperature, HR, and RR in a typical case of comparing physiological events in the control ward and Lab-1 during a 5-day period from the very hot month of April 2013. The two babies being compared were water-sponged several times during the day due to EFS attack whilst they remained in the control ward. However when one of them was transferred to Lab-1 on April 11 just before midnight, this baby recovered and never had any need for water sponging again for the rest of the comparing period unlike its counterpart that remained in the control ward (Figure 9).

The heat exchanger resulted in average water flow rate of 2.3 litres per minute. There was observable difference in the relative coolness of Lab-1 with the heat exchanger operation, especially just before the sun began to set at 6 pm. Average inlet/outlet temperatures of water through the exchanger are shown in Table 1.

## 4. Discussion

The present study has sought to create and validate a method of building a sustainable weather-proof neonatal nursing environment that can curb morbidity due to tropical evening-fever syndrome (EFS). At study inception we hypothesised that a naturally cooled nursing environment with minimised heat-impact of the sun would guarantee effective incubator performance in maintaining desired incubation set-points without externally influenced overheating. We also hoped that such thermal-friendly environment would guarantee a more stable nursing of open-cot babies within the environment with lower frequency of hyperthermia due to EFS. An earlier study demonstrated that neonates readily tend to assume body temperatures similar to their environmental room temperatures when exposed to these [1]. This is consequential to the classical procedures of maintaining effective neonatal warming in a cold environment using appropriately designed wrapping suits for full-term neonates and adequately controlled incubator microenvironments for preterm neonates [12, 13].

Preterm neonates exhibit higher ratio of skin-surface-area to body-mass as compared to full-term neonates. This delimits the preterm neonate's ability to autoregulate its body temperature when exposed to a room temperature outside the physiological range. Therefore, EFS can fundamentally set in when environmental room temperature exceeds the acceptable physiological body upper limit of 37.4°C. The present study created Lab-1 and Lab-2, testing their respective abilities to maintain room temperatures that were well below this physiological limit even with prevailing extreme heat intensity from the sun. Our Lab-1 results have shown that by carefully integrating some factors as considered in this study, a naturally cooled environment could be guaranteed. Lab-2 was relatively warmer than Lab-1 because this lacked the additional cooling effect the underground floor/walls provided in Lab-1. The control ward's room temperatures readily exceeded this physiological upper limit as can be seen from Figure 6. This consequently led to a very frequent manifestation of EFS, incubator overheating, and general un-wellness of babies in this ward.

In general, the room temperatures of all the wards during the early hours of the day were far below the hypothermic threshold of 36.5°C and at times as low as 26°C. This condition would normally require the incubators to operate at high set-points to supply extra heating for the preterm neonates while cot-babies were given adequate extra wrapping. The room temperatures of our control ward changed very rapidly with the appearance of the sun and its rising intensity as the day progressed into the afternoon. This rapid increase of room temperature resulted in additional uncontrollable heating that would soon overheat the cot-babies, the incubator, and its occupant. In most cases the attending nurses were unaware of the hyperthermic babies until the next vital-signs check that happened only once every 4 hours (6 am, 10 am, 2 pm, 6 pm, and 10 pm), with 2 am being verily inconsistent. The only exception to this was in cases where the uncomfortable babies reserved any energy to cry or show signs of unrest or the incubators produce overheat alarm. In extreme cases complete switch-off of incubator heating would not remove the problem as the sun singularly supplied enough energy to warm the nursery beyond 38°C. This results in more and more babies being given the ultimate solution of “temporary comfort” of water sponging. We do not yet know of the consequences of such sudden quench of high fever on the neonates as this has not been studied. However, such practice of sudden temperature crash from hyperthermia to hypothermia and a quick buildup back to hyperthermia without any obvious thermal stability creates a condition of neonatal “thermal shock” that could readily initiate apnoeic attacks.

This extreme complication was however not observed in the “test wards” due to their relative coolness and more sluggish rate of temperature increases as compared to the control ward. Lab-1 maintained an average temperature-gain rate of 0.75°C/hr to average maximum peak of 33 ± 1°C as compared to the control ward at average rate of 1.63°C/hr to average maximum peak of 39 ± 1°C. This situation guaranteed a more stable and relatively cooled Lab-1 enabling the incubators to solely control their neonatal microenvironments, maintaining their operational set-points for the entire day.

The operational effectiveness of the new lab designs in maintaining such coolness in a life-size nursery unit becomes more believable when Lab-1 relative disadvantages are considered. Lab-1, for example, was only a small room less than 12% of the area size of the control, with only 2 windows and 1 door. Hence, this lacked the airy advantage that could possibly aid the cooling of the control.

During the period of this experimentation, nurses reported instances when incessant hyperthermia would have led doctors to suspect a disease process and hence commenced antibiotic treatment as a matter of procedural practice. Instead, some of the nonparticipating patients were temporarily moved to Lab-1 (one at a time due to lack of cot space) and the patients quickly recovered, similar to the example in Figure 9. This clearly demonstrated no presence of disease for which the use of antibiotic would have led to complications that could be capable of claiming the life of such baby. Lab-1 clearly eliminated EFS but still required well-trained nurses for effective dynamic regulation of the incubator to determine the appropriate set-point that could stabilise baby within the safe zone of 36.5°C–37.4°C. Baby could become hypothermic if lower-than-required incubator set-point was maintained. This explained why “baby control-Lab-1” showed signs of hypothermia during the first few hours in Lab-1 (Figure 9). The comparative effectiveness of the labs can be appreciated from Figure 6. This revealed that the control ward was overheated far above the physiologically acceptable neonatal upper limit temperature of 37.4°C. This explains why so many water-sponging events were recorded in the control on the one hand (Figure 8). On the other hand, the temperature peak values of the 2 labs never rose up to this physiological limit, resulting in the relative neonatal stability as depicted by Figure 8. We can therefore conclude that neonatal EFS can be reduced by correcting an existing SCBU building using the specifications of Lab-2 or where the funds could be afforded, eradicated by the construction of entire new structure with the specifications of Lab-1. The cost of erecting Lab-1 patterned structure might be slightly higher than that of the control ward based on projected bill-of-quantity; however, this could be quickly offset by the running cost of the air-conditioning units in control ward during operation. At experimented heat extraction factor of 2.2°C/unit-time the heat exchanger time of operation would be between 3 pm and 4 pm for best result.

Further study would investigate a possible link of disease appearance to the presence of EFS. We do not understand why EFS resulted in extremely low RR sign in some patients and extremely high in others whilst HR remained slightly high. This might be indicative of some other variables for which further investigation is required. El-Radhi et al. (2009) noted that tachycardia is a sign of fever; this is consistent with the high RR in our observation [14]. However, the cases of lower RR might as well be associated with the “thermal-shock” effect explained earlier and hence a gradual approach of apnoea. A foreseeable limitation to the application of the present proposals (i.e., Lab-1) is in the event of flooding. Since such nursery design is likely going to be the lowest floor in the hospital complex, adequate allowance to prevent any possible occurrence of flooding must be incorporated in the design. Adequate precautions should be taken to avoid development of wall cavities that could lead to stagnation of leaking water as this could become breeding grounds for mosquitos and dampness with possible consequences of malaria or other infections.

## Figures and Tables

**Table 1:** Heat exchanger temperatures during operation.

Average temperature	12 noon	3 pm	6 pm
Inlet (°C)	32.4	32.6	33.4
Outlet (°C)	33.5	34.8	34.9
Heat extraction factor/unit time	1.1°C	2.2°C	1.5°C

Inlet temperature of water was measured at the entry valve point just before this entered building. Outlet temperature was measured at the point of exit immediately after leaving the building (please see Figures 2(a) and 2(b)).
